# Supplementary material for: Ultrasmall single-layered NbSe2 nanotubes flattened within a chemical-driven self-pressurized carbon nanotube
Source: Nat Commun. 2024 Jan 11;15:475. doi: 10.1038/s41467-023-44677-y (PMC10784551; doi:10.1038/s41467-023-44677-y)
Supplement: Supplementary file 1 — Supplementary Information [file 41467_2023_44677_MOESM1_ESM.pdf]

Supplementary Information

Ultrasmall single-layered $NbSe_2$ nanotubes flattened within a chemical-driven self-pressurized carbon nanotube

Authors: Yaxin Jiang[1], Hao Xiong[1], Tianping Ying[2], Guo Tian[1], Xiao Chen[1,3]*, Fei Wei[1,3]*

Affiliations:

[1]Beijing Key Laboratory of Green Chemical Reaction Engineering and Technology, Department of Chemical Engineering, Tsinghua University, Beijing 100084, China.

[2]Beijing National Laboratory for Condensed Matter Physics, Institute of Physics, Chinese Academy of Sciences, Beijing 100190, China.

[3]Ordos Laboratory, Ordos, Inner Mongolia 017000, China.

*Corresponding author. Email: chenx123@mail.tsinghua.edu.cn (X.C.); wf-dce@mail.tsinghua.edu.cn (F.W.)

Supplementary Notes:

Equivalent diameter of radial-deformed $NbSe_2$ and carbon nanotube

Although $NbSe_2$ flat tubes do not necessarily fill the entire nanotube, their length can reach hundreds of nanometers. After thinning to 60 nm thick, the cross-sectional atomic structure of CNT and $NbSe_2$ can be clearly distinguished by the ADF-STEM imaging. Therefore, by measuring the perimeter (C) of the Nb atom or the carbon atom, the equivalent diameter of a circle with the same perimeter can be calculated as $d=C/\pi$.

Analysis of minor-axis spacing of $NbSe_2$ flat tube

Similarly, the minor-axis spacing which is defined as the distance between the Nb atoms of the upper and lower walls in the minor-axis direction can be measured by the profile analysis. The selected area generally passes through the center of the ellipse circle to reduce the experimental error.

Native strain of CNT host arising from internal $NbSe_2$ flat tube

The details of the hexagonal distortion in the tube cross-section play an important role in determining the frequency of the Raman G^+ and 2D modes in CNTs. Further, the native strain (ε) can be estimated by using an equation,

$$\Delta\omega(\mathrm{cm}^{-1}) = A\ \varepsilon \qquad (1)$$

where $A \approx 8.6$ and 37.3 cm^{-1} per % strain for the G^+ and 2D bands of an individual CNT under 532 nm laser illumination, respectively. When employing 633 nm laser resonance conditions, A is slightly increased to 8.8 cm^{-1}. Accordingly, the strains of 0.4-1.2% were calculated from the shifts of the Raman-mode frequencies.

On the other hand, the native strain of the CNT can be obtained from cross-sectional STEM images. The equivalent diameter ($2R$) and half-length of major-axis (R_{major}) and minor-axis (R_{minor}) of the CNT host were obtained after detailed measurements, and then the strain along the major-/minor- axis direction ($\varepsilon_{major/minor}$) can be calculated as

$$\varepsilon_{major}=\frac{R_{major}-R}{R},\ \varepsilon_{minor}=\frac{R_{minor}-R}{R} \qquad (2)$$

Pressure effects on the calculated formation energy

The $NbSe_2$ nanostructures were experimentally demonstrated to be pressurized in CNTs at the gigapascal level, therefore we calculated and compared their formation energy under applied pressure (4-20 GPa) in addition to vacuum conditions. As shown in Supplementary Fig. 17, the formation energies of the different $NbSe_2$ configurations (n=10) changed upon applying pressure. A certain pressure will make the tubular structure, whether circular or flat, more stable, and vice versa for the lamellar structure. However, the fluctuations in formation energy relative to vacuum conditions range from 10^{-5} to 10^{-7} eV per atom, and remain almost constant with increasing pressure above 4 GPa. Further, the flat tube still features the lowest formation energy at all calculated pressures at n = 12, which proves to be the most energetically stable configuration (Supplementary Fig. 18). Therefore, the pressurized condition has little effect on the calculated formation energy. We then compared the formation energy for different numbers of cross-sectional $NbSe_2$ units under applied 4 GPa pressure. The calculated results are quite similar to Fig. 3a, confirming that the flat tube is the most stable configuration in a certain diameter range (Supplementary Fig. 19).

DFT-calculated energetic stability of $NbSe_2$ circular tube

Armchair circular tubes with cross sections of different $NbSe_2$ units (*n*, *n*=6-24) were constructed as initial structures, with atomic positions derived from the bulk solid, which were then fully relaxed by minimizing the total energy. Because of the three atomic planes of their walls, the nanotube exhibits high bending rigidity. The relaxed circular tube unravels spontaneously at *n*=6, as shown by the absence of closed terminations. While the structure was relaxed into a flat tube at *n*=8, where its edges are indeed closed. Only for n>10, the tube with an intrinsic wall thickness could maintain a circular tubular structure (Supplementary Fig. 16). However, the optimized smallest $NbSe_2$ circular tube (*n*=10) had an average elongation of 2.43% for the outer bond and 0.98% compression for the inner bond, and hence significant formation energy.

Correlation strength given by the Luttinger parameter, *K*

In a Luttinger liquid, the strength of the interaction between electrons can be approximated by a single dimensionless parameter, the Luttinger parameter *K*,

$$K \approx \left[1+\frac{W(q=0)}{\pi\hbar v_F}\right]^{1/2} \qquad (3)$$

where $W(q=0)$ is the charging energy of the tube which is susceptible to the dielectric environment, and $\pi\hbar v_F$ is the inverse density of states for 1D Dirac electrons that depends on the detail of the electronic band dispersion. When the long-range Coulomb interaction between electrons exceeds their kinetic energy, resulting in $K<1/2$, while $K<1/4$ is further defined as the limit of very strong interactions[1,2].

For an SWNT with four conducting modes at E_F, as a power law in temperature with the scaling exponent α, related to the Luttinger parameter as[3],

$$\alpha=(K+K^{-1}-2)/8 \qquad (4)$$

The deriving Luttinger parameters are distinct for different systems, where $K_{CNTs} = 0.29$, $K_{NbSe3@CNT} = 0.32$, and $K_{NbSe2@CNT} = 0.23$, respectively. Although *K* all fall in the strong coupling limit, the interaction is tunable either up or down, depending on the encapsulated species. And for $NbSe_2$, it reaches even the very strong interactions range.

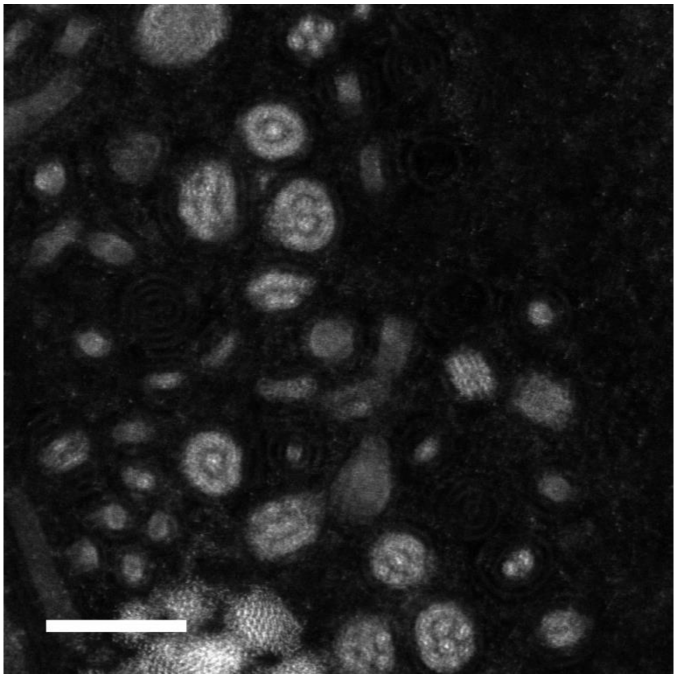

Supplementary Figure 1. **Representative cross-sectional HAADF-STEM images of the $NbSe_3$ chains encapsulated within CNTs**. It shows different close-packing patterns associated with the inner diameters of the CNT host. Scale bars: 5 nm.

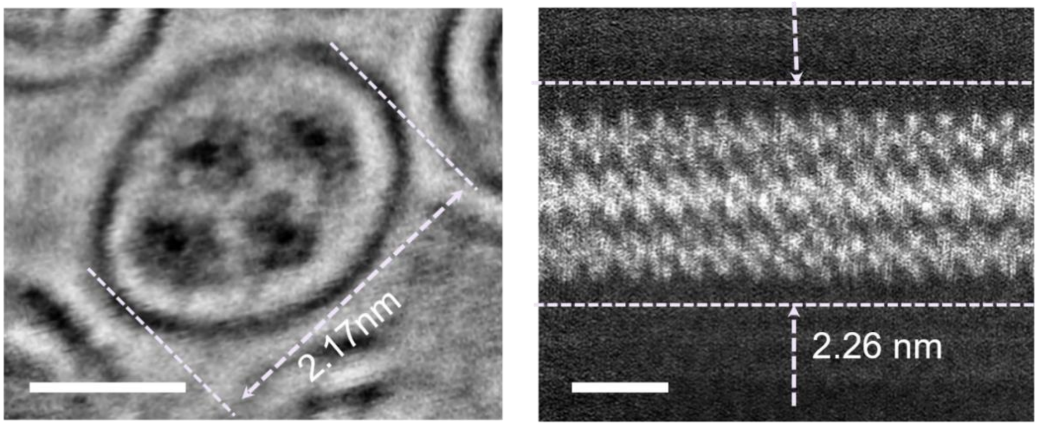

Supplementary Figure 2. **Cross-sectional BF-STEM image (left) of four $NbSe_3$ chains**. These chains are encapsulated in a CNT with a major-axis length of 2.17 nm, which can correspond to the paralleling $NbSe_3$ triple-chain structure in a CNT with a similar inner diameter (2.26 nm) from the side-view projection ADF-STEM image (right). Scale bars: 1 nm.

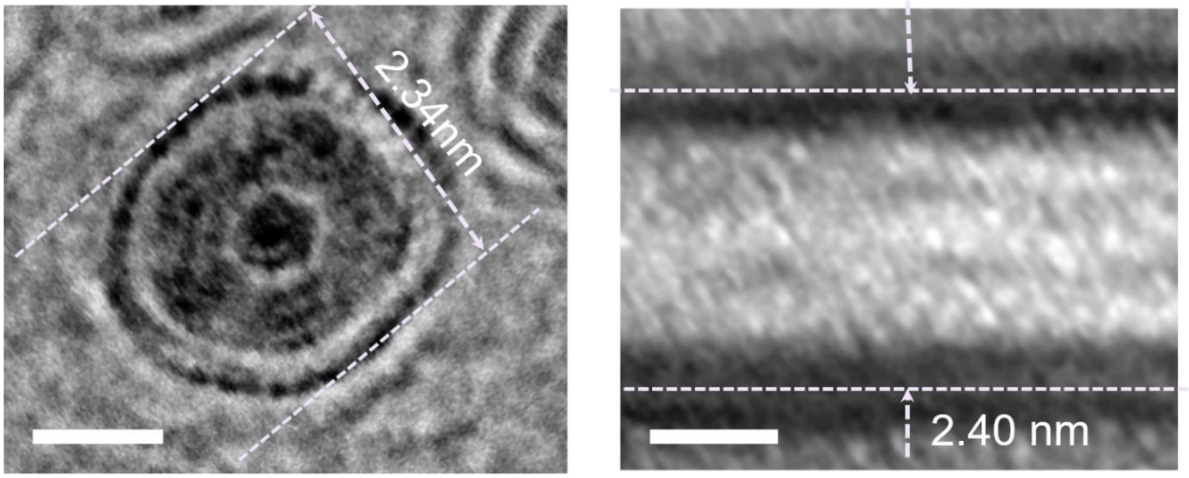

Supplementary Figure 3. **Cross-sectional BF-STEM image (left) of ~8 $NbSe_3$ chains.** These chains are encapsulated in a CNT with an inner diameter of 2.34 nm, corresponding to the spiraling $NbSe_3$ triple-chain in 2.4 nm CNT (right). Scale bars: 1 nm.

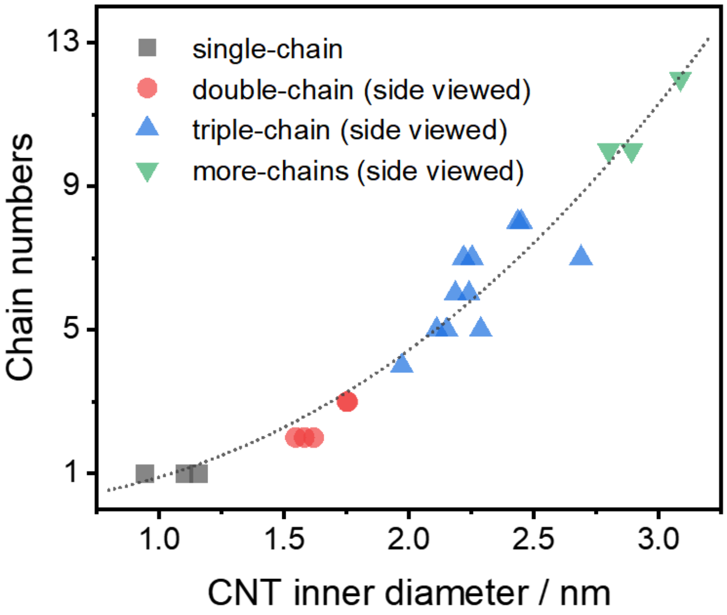

Supplementary Figure 4. **The actual chain numbers of the encapsulated $NbSe_3$ in the cross-sectional STEM images versus the inner diameter of CNTs.**

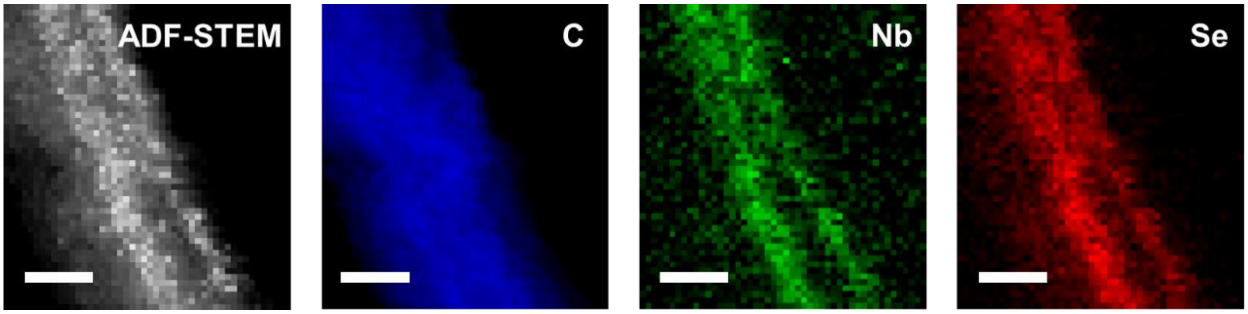

Supplementary Figure 5. **ADF-STEM of $NbSe_2$@CNT and corresponding chemical elemental mapping of C, Nb, and Se by Electron Energy Loss Spectroscopy (EELS).** Scale bar: 2 nm.

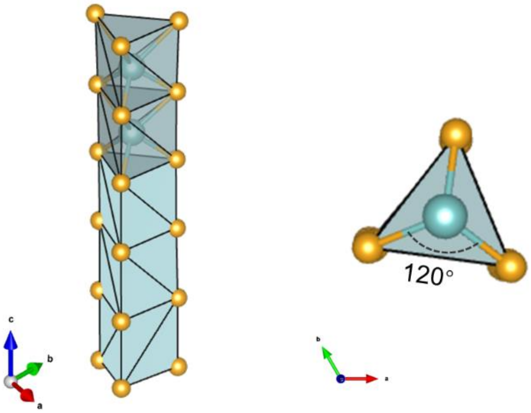

Supplementary Figure 6. **Optimized atomic models of encapsulated $NbSe_3$ single-chain**. The models are optimized based on DFT calculations (the CNT host is not shown for clarity), which presents the identical end-viewed Se-Nb-Se projection angle different from that of its bulk counterpart.

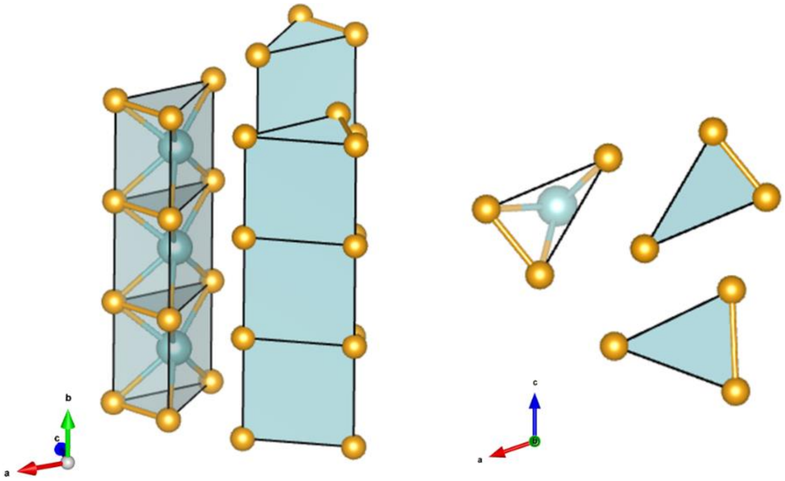

Supplementary Figure 7. **Atomic models of $NbSe_3$ chain-like bulk phase from the end-view projection.**

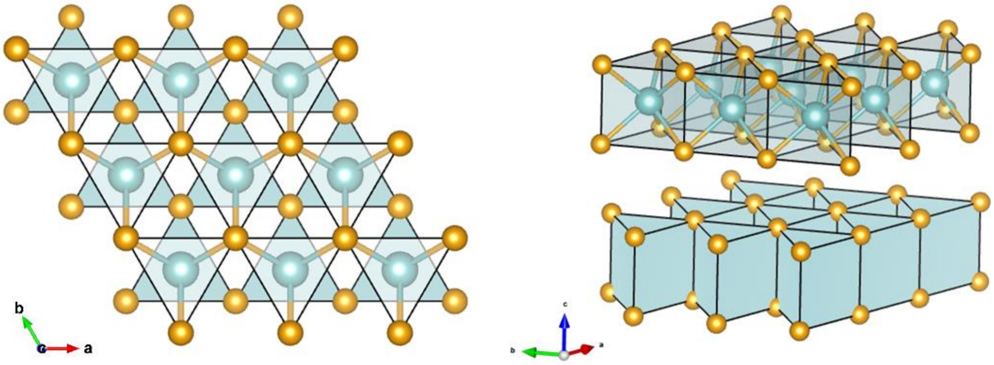

Supplementary Figure 8. **Atomic models of $NbSe_2$ lamellar bulk phase from end- and plane-view projection.**

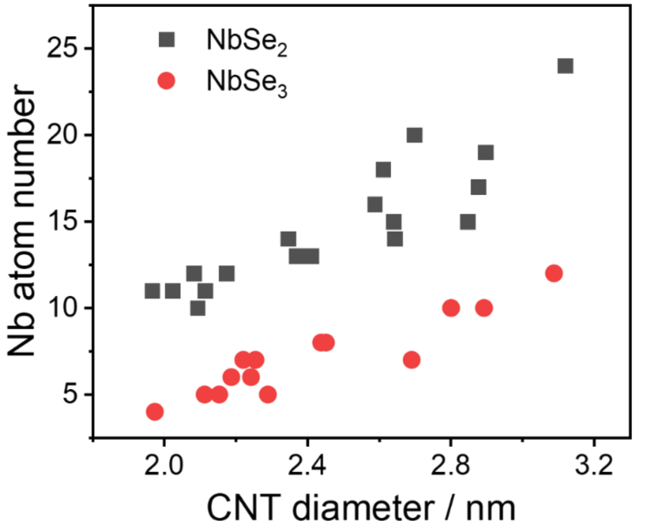

Supplementary Figure 9. The Nb atom numbers of the encapsulated $NbSe_2$ and $NbSe_3$ in the cross-sectional STEM images versus the inner diameter of CNTs.

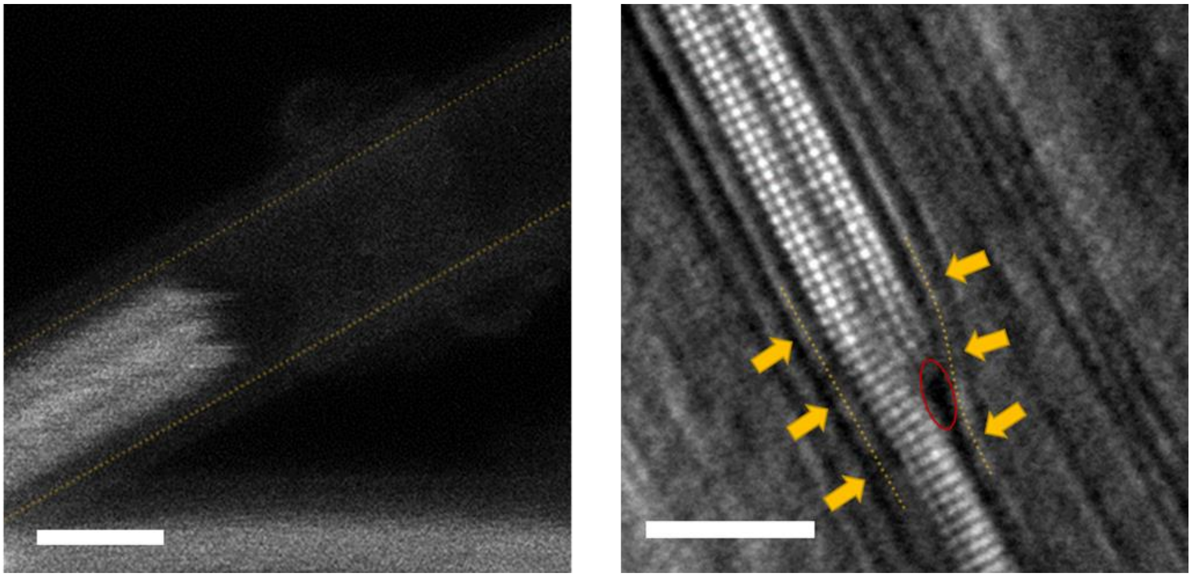

Supplementary Figure 10. **Side-viewed ADF-STEM image of NbSe3@CNTs and BF-STEM image of NbSe2@CNTs, respectively**. Scale bar: 2 nm. It shows the different contractions of the CNT capsule after the deselenization reaction due to the different expansion degrees of the internal $NbSe_2$, applying unequal pressures and thus pulling out voids in the red ellipse.

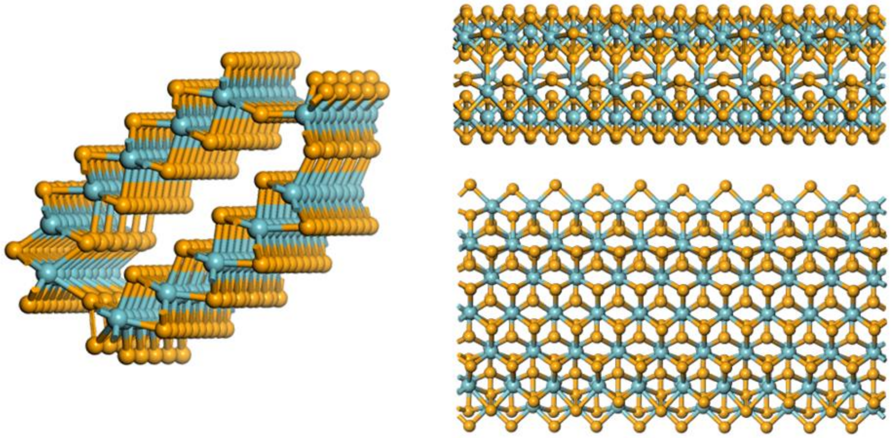

Supplementary Figure 11. **Atomic model of $NbSe_2$ flat tube**. It is fully relaxed by DFT calculations, which matches the experimental images from the side-view projection in Fig. 1.

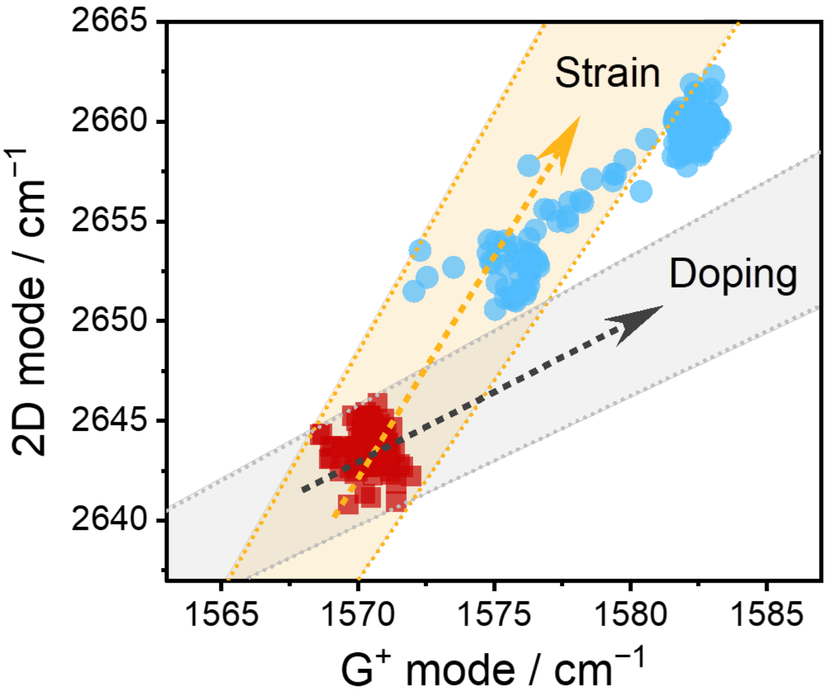

Supplementary Figure 12. **Decoupling of Raman modes for samples taken at 532 nm laser excitation wavelength.** It indicates these shifts are mainly determined by strain effect.

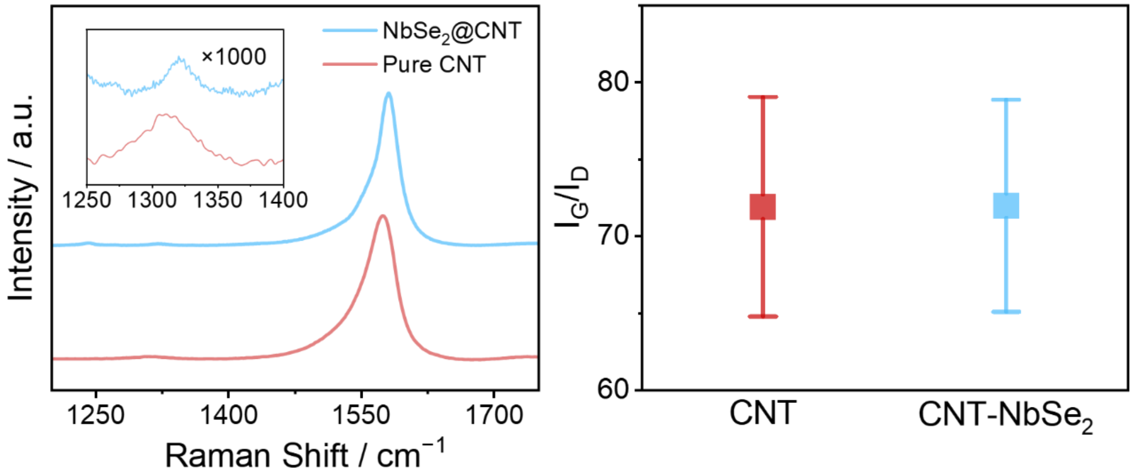

Supplementary Figure 13. Comparison of Raman spectra of CNTs and NbSe2@CNT samples. The experiments were taken under 633 nm wavelength laser (left), where the intensity is normalized at the G mode. After magnifying 1000 times, the D-mode intensity is still very weak. The intensity ratio of G-mode and D-mode (right) after encapsulation and transformation remains at ~70, suggesting the structural integrity of the CNT with few and no increase in defects on its surface.

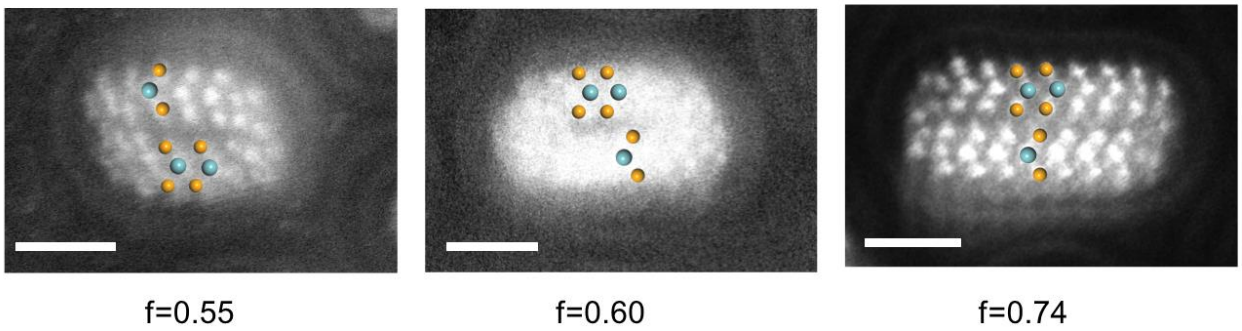

Supplementary Figure 14. **Representative cross-sectional ADF-STEM images of $NbSe_2$ flat tube within CNTs of different diameters, with atomic models overlaid.** The ellipticity of the $NbSe_2$ flat tube increases from 0.55 to 0.74, which corresponds to the transition from near-circular to elliptical. Scale bars, 1 nm.

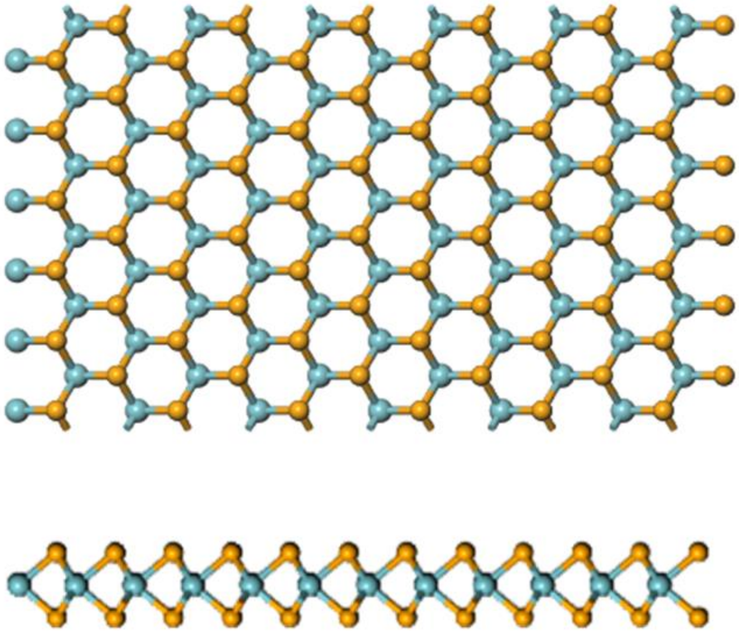

Supplementary Figure 15. Representative atomic model of $NbSe_2$ single-layer with *n*=12.

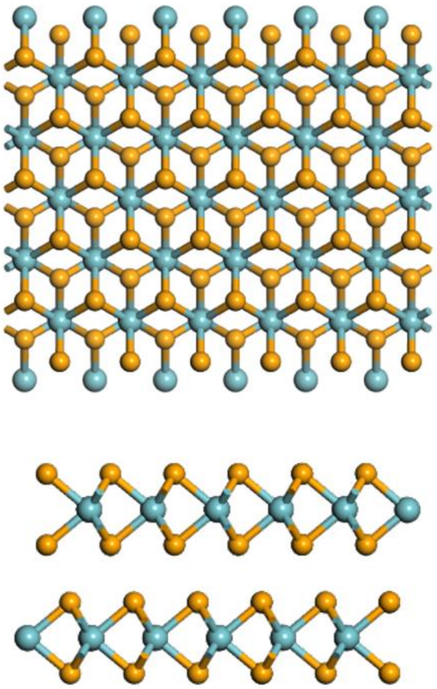

Supplementary Figure 16. Representative atomic model of $NbSe_2$ double-layer with n=12.

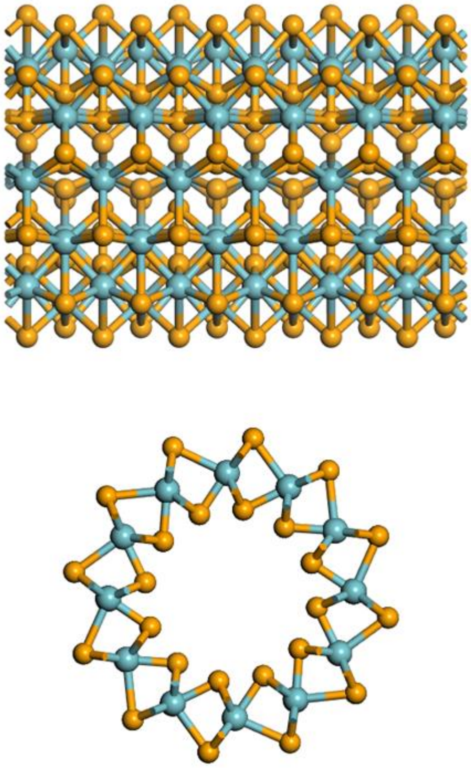

Supplementary Figure 17. Representative atomic model of NbSe2 circular tube with n=12.

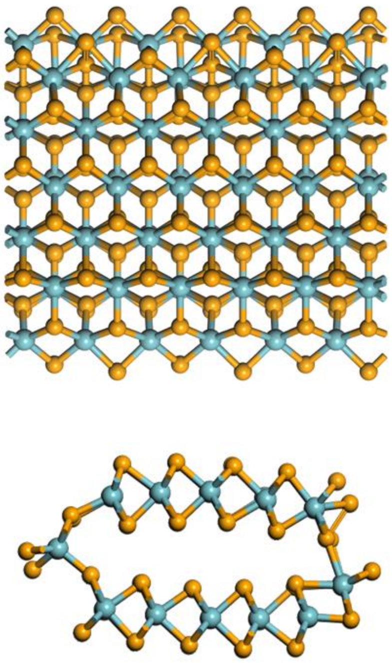

Supplementary Figure 18. **Representative atomic model of $NbSe_2$ flat tube with n=12.**

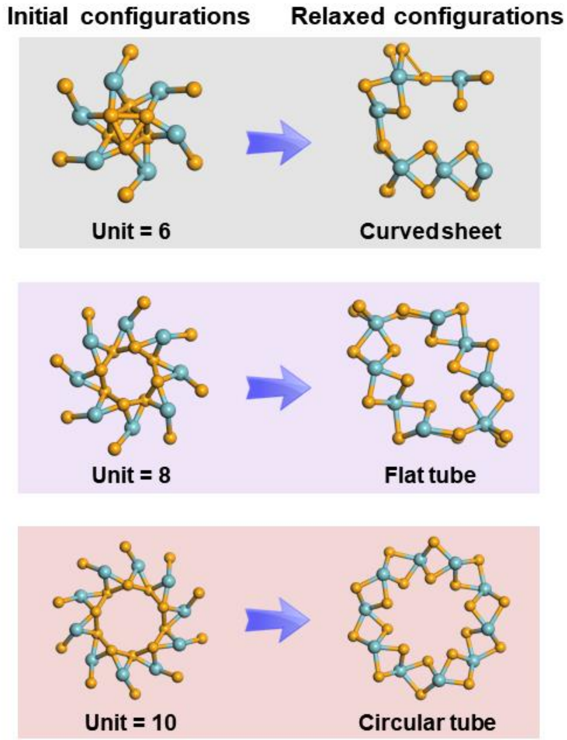

Supplementary Figure 19. **Cross-sectional view of circular tubular structure with different n (n=6-8).** The Nb and Se atoms in initially and the corresponding relaxed configurations are in blue and yellow. DFT calculations show the formation of other configurations after a geometry optimization, indicating the instability of the ultranarrow circular tube.

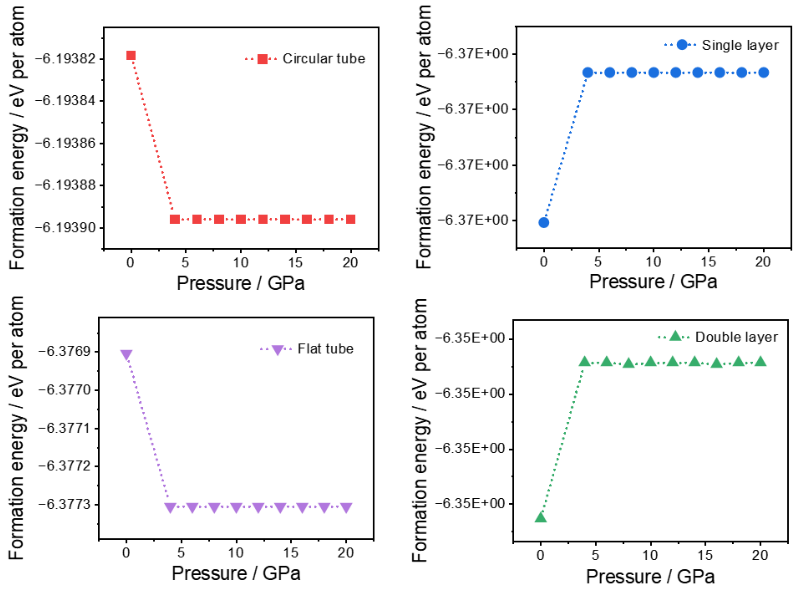

Supplementary Figure 20. **Formation energy calculated by applying different pressures (4-20 GPa) at n=10.**

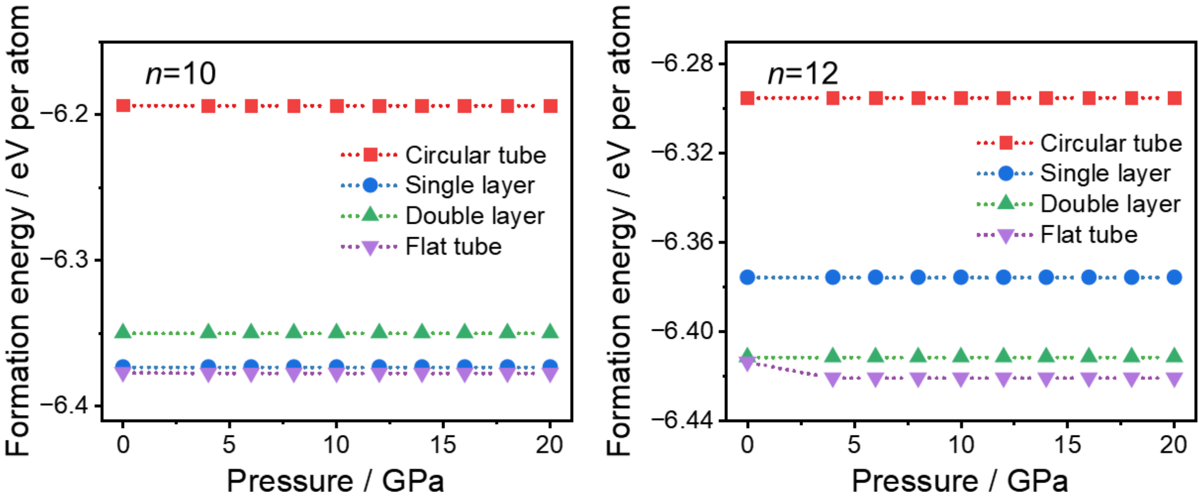

Supplementary Figure 21. **Comparison of formation energy of varied $NbSe_2$ configurations under different pressures at n=10 (left), 20 (right).**

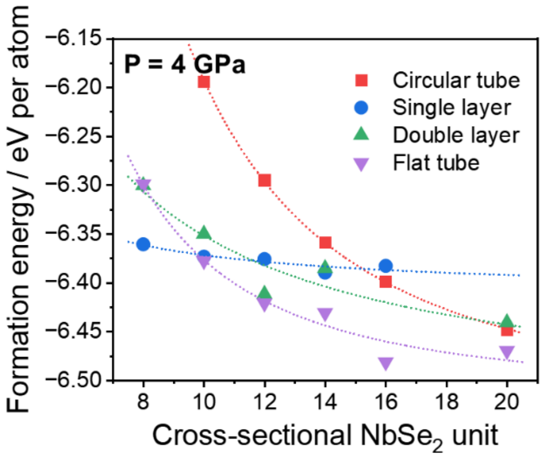

Supplementary Figure 22. **Forming energy of varied $NbSe_2$ configurations as a function of n under 4 GPa.**

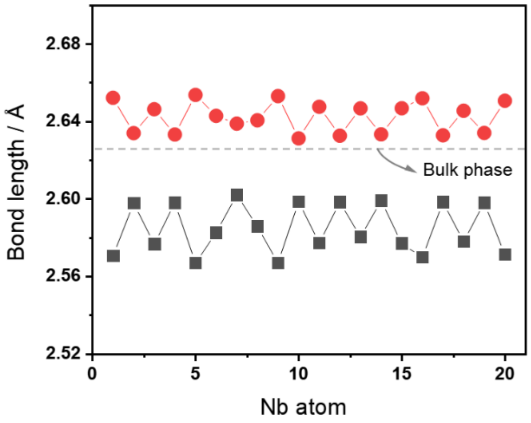

Supplementary Figure 23. **Bond length distribution of Nb-Se bonds in the $NbSe_2$ circular tube at n=20.** The red circles represent the length of outer Nb-Se bonds, while the black rectangles show the inner Nb-Se bond length.

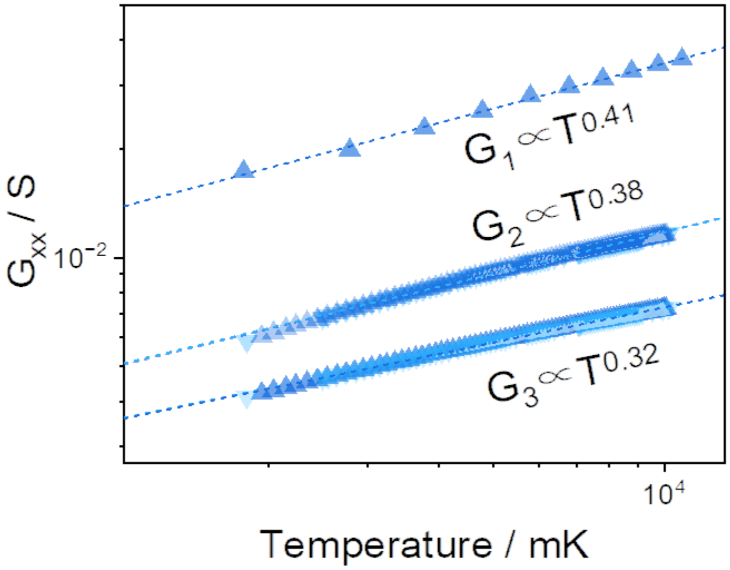

Supplementary Figure 24. Power law relation of conductance G with temperature (G ~ Tα) for three $NbSe_2$@CNT samples from 2-100 K. It shows a similar fitting exponent α = 0.32-0.33 from the fitting curves.

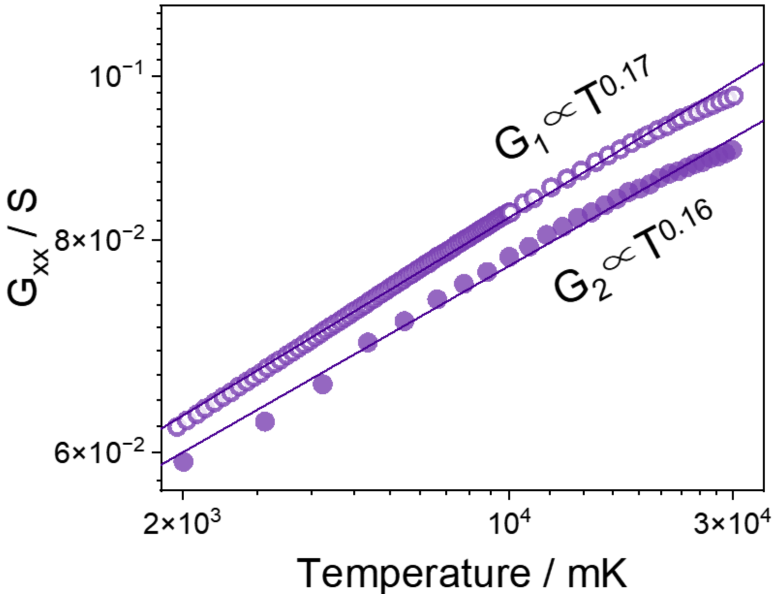

Supplementary Figure 25. Power law relation of conductance G with temperature (G ~ Tα) for two $NbSe_3$@CNT samples from 2-30 K. It shows a similar fitting exponent α = 0.16-0.17 from the fitting curves.

Sample	Element	Atomic%	Peak binding energy / eV	Assignments
CNT	C	99.52	284.78<br>285.49	C-C<br>C-O
	Nb	0.04	\	\
	Se	0.44	\	\
$NbSe_2$@CNT	C	96.66	284.81<br>285.58	C-C<br>C-O
	Nb	0.99	204.19<br>206.98<br>207.46<br>210.13	Nb^{4+} $3d_{5/2}$<br>Nb^{4+} $3d_{3/2}$<br>Nb^{5+} $3d_{5/2}$<br>Nb^{5+} $3d_{3/2}$
	Se	2.34	54.11<br>56.02	Se $3d_{5/2}$<br>Se $3d_{3/2}$
$NbSe_3$@CNT	C	92.8	284.78<br>285.43	C-C<br>C-O
	Nb	1.4	204.44<br>207.14<br>207.24<br>210.10	Nb^{4+} $3d_{5/2}$<br>Nb^{4+} $3d_{3/2}$<br>Nb^{5+} $3d_{5/2}$<br>Nb^{5+} $3d_{3/2}$
	Se	5.8	53.68<br>55.84	Se $3d_{5/2}$<br>Se $3d_{3/2}$
$NbSe_2$ bulk phase	Nb	\	203.2<br>205.8	Nb^{4+} $3d_{5/2}$<br>Nb^{4+} $3d_{3/2}$
	Se	\	54.2<br>55.1	Se $3d_{5/2}$<br>Se $3d_{3/2}$
$NbSe_3$ bulk phase	Nb	\	203.6<br>206.4	Nb^{4+} $3d_{5/2}$<br>Nb^{4+} $3d_{3/2}$
	Se	\	54.6<br>55.5	Se $3d_{5/2}$<br>Se $3d_{3/2}$

Supplementary Table 1. Summary of the atomic composition and core-level assignments obtained from the fitting of the XPS spectra.

Species	ΔE/eV	G-E/eV (thermal corrections)	ΔG/eV
$NbSe_3$	-23.44	/	-23.44
$NbSe_2$	-20.65	/	-20.65
Se	-3.50 (0 K)	-1.10 (973K)	-4.59 (973K)

Supplementary Table 2. Gibbs free energy of $NbSe_3$, $NbSe_2$ and Se bulk materials obtained from DFT calculations.

Temperature /K	Deselenization reaction : $NbSe_3 \rightleftharpoons NbSe_2 + Se$	
	ΔG/eV	ΔG/kJ mol^{-1}
0	-0.71	-68.44
973	-1.81	-173.32

Supplementary Table 3. Gibbs free energy difference of deselenization reaction.

Supplemeantary References

1. Li, T. *et al.* Observation of a Helical Luttinger Liquid in InAs/GaSb Quantum Spin Hall Edges. *Physical Review Letters* **115**, doi:10.1103/PhysRevLett.115.136804 (2015).
2. Ziani, N. T., Crepin, F. & Trauzettel, B. Fractional Wigner Crystal in the Helical Luttinger Liquid. *Physical Review Letters* **115**, doi:10.1103/PhysRevLett.115.206402 (2015).
3. Braunecker, B., Bena, C. & Simon, P. Spectral properties of Luttinger liquids: A comparative analysis of regular, helical, and spiral Luttinger liquids. *Physical Review B* **85**, doi:10.1103/PhysRevB.85.035136 (2012).
